# Automated subcortical volume estimation from 2D MRI in epilepsy and implications for clinical trials

**DOI:** 10.1007/s00234-021-02811-x

**Published:** 2021-10-18

**Authors:** Daniel Brownhill, Yachin Chen, Barbara A. K. Kreilkamp, Christophe de Bezenac, Christine Denby, Martyn Bracewell, Shubhabrata Biswas, Kumar Das, Anthony G. Marson, Simon S. Keller

**Affiliations:** 1 Department of Pharmacology and Therapeutics, Institute of Systems, Molecular and Integrative Biology, University of Liverpool, Liverpool, UK; 2 Neurological Science, Clinical Sciences Centre, Aintree University Hospital, Lower Lane, Liverpool, L9 7LJ UK; 3 Department of Clinical Neurophysiology, University Medicine Göttingen, Göttingen, Germany; 4 The Walton Centre NHS Foundation Trust, Liverpool, UK; 5 Schools of Medical Sciences and Psychology, Bangor University, Bangor, UK

**Keywords:** Epilepsy, Two-dimensional segmentation, Subcortical, Automatic segmentation

## Abstract

**Purpose:**

Most techniques used for automatic segmentation of subcortical brain regions are developed for three-dimensional (3D) MR images. MRIs obtained in non-specialist hospitals may be non-isotropic and two-dimensional (2D). Automatic segmentation of 2D images may be challenging and represents a lost opportunity to perform quantitative image analysis. We determine the performance of a modified subcortical segmentation technique applied to 2D images in patients with idiopathic generalised epilepsy (IGE).

**Methods:**

Volume estimates were derived from 2D (0.4 × 0.4 × 3 mm) and 3D (1 × 1x1mm) T1-weighted acquisitions in 31 patients with IGE and 39 healthy controls. 2D image segmentation was performed using a modified FSL FIRST (FMRIB Integrated Registration and Segmentation Tool) pipeline requiring additional image reorientation, cropping, interpolation and brain extraction prior to conventional FIRST segmentation. Consistency between segmentations was assessed using Dice coefficients and volumes across both approaches were compared between patients and controls. The influence of slice thickness on consistency was further assessed using 2D images with slice thickness increased to 6 mm.

**Results:**

All average Dice coefficients showed excellent agreement between 2 and 3D images across subcortical structures (0.86–0.96). Most 2D volumes were consistently slightly lower compared to 3D volumes. 2D images with increased slice thickness showed lower agreement with 3D images with lower Dice coefficients (0.55–0.83). Significant volume reduction of the left and right thalamus and putamen was observed in patients relative to controls across 2D and 3D images.

**Conclusion:**

Automated subcortical volume estimation of 2D images with a resolution of 0.4 × 0.4x3mm using a modified FIRST pipeline is consistent with volumes derived from 3D images, although this consistency decreases with an increased slice thickness. Thalamic and putamen atrophy has previously been reported in patients with IGE. Automated subcortical volume estimation from 2D images is feasible and most reliable at using in-plane acquisitions greater than 1 mm x 1 mm and provides an opportunity to perform quantitative image analysis studies in clinical trials.

**Supplementary Information:**

The online version contains supplementary material available at 10.1007/s00234-021-02811-x.

## Introduction

Automated segmentation and volume estimation of brain structures on magnetic resonance (MR) images offer a substantial advantage in epilepsy research. Quantitative MRI methods can be incorporated into clinical evaluation of patients with refractory epilepsy who are being considered for neurosurgery, and volumetric data can be included in prognostic models of treatment outcome in clinical trials. Nowadays, automated quantitative MRI techniques are preferred over manual methods given the superior time efficiency and improved reproducibility[1], while retaining consistency with gold-standard manual delineation methods for volume estimation of subcortical volume [2, 3] and high levels of scan-rescan reproducibility [4]. Automated methods, however, are traditionally applied to three-dimensional (3D) image data that have isotropic voxels and full head coverage. This can be problematic for clinical trials as MRI data may be obtained from non-specialist centres; image data collected here may be two-dimensional (2D), non-isotropic and lacking full spatial head coverage. Clinical trials generate a wealth of patient-relevant data which, combined with quantitative image analysis, could potentially provide meaningful insights into mechanisms, biomarkers and prognostic factors in neurological disorders. Some epilepsy clinical trials have been unable to investigate the prognostic significance of quantitative imaging data given the lack of 3D MRI data and have been restricted to binomial classifications of lesional or non-lesional imaging findings from non-specialist radiology departments [5, 6]. The ability to automatically segment and quantify volumes from 2D MR images routinely acquired in non-specialist hospitals may therefore provide important information in prognostic models of treatment outcome in clinical trials.

Modification of 2D MR images for automated segmentation and subcortical volume estimation has already been performed in studies of multiple sclerosis (MS) [7, 8]. This modification has included interpolation from a non-isotropic to an isotropic resolution and image cropping to maintain uniformity between patient images [7]. These studies indicate that segmentation and volume estimation of subcortical regions is less challenging than segmentation and parcellation of the cerebral cortex [7, 8]. Volume estimates of subcortical structures have been reported to show respectable agreement between 2 and 3D MR images of the same patients with MS when automatically segmented with the freely available and widely used FIRST (FMRIB Integrated Registration and Segmentation Tool) [7] that is part of FMRIB Software Library (FSL) [9]. FIRST is a model-based segmentation and registration tool generated from manually delineated images for segmentation of the caudate nucleus, globus pallidus, hippocampus, nucleus accumbens, putamen and thalamus, brainstem and amygdala [9]. FIRST applied to 3D images has frequently been used to estimate the volume of subcortical structures in patients with epilepsy [10–14]. In patients with idiopathic generalised epilepsy (IGE), volume reduction has been reported in several subcortical structures, most frequently in the thalamus [2, 15–25] and putamen [17–19, 26, 27]. This thalamic and putamen atrophy fits well with the known pathophysiology of the disorder [28–31]. Therefore, IGE is an excellent model for the evaluation of consistency of subcortical volume alterations between segmentations derived from 2 and 3D images.

In the present study, we aim to determine the consistency of subcortical volume estimation from 2 and 3D images by altering a modified FSL FIRST method in patients with IGE and healthy controls. In the first part of the study, we examine the degree of consistency between segmentations obtained from 2 and 3D images from the same scanning session. In the second part of the study, we investigate whether volume changes in subcortical structures in patients with IGE are consistently observed using 3D and 2D image segmentations.

## Methods

### Participants

We recruited 31 patients with a diagnosis of IGE from the Walton centre NHS Foundation Trust, Liverpool, UK and informed written consent was obtained for all participants (local research ethical committee reference 14/NW/0332). All patients were diagnosed with IGE by a qualified neurologist using the ILAE classification [32], and based on patient history, semiological features, and EEG. There were no potentially epileptogenic or incidental brain lesions on diagnostic MRI. Thirty-nine age and sex matched healthy controls were also recruited. Demographic information for the cohort is summarised in Table 1.

**Table 1:** (a) Clinical characteristics of patients. Year as the unit for age, onset and duration. Onset indicates the age of onset of epilepsy. F = female; M = male; REF = refractory; FH = family history; PS = photosensitive; ASM = anti-seizure medication (daily dose in milligram); AS = absence seizures; GTCS = primary generalized tonic–clonic seizures; MS = myoclonic seizures. (b) Demographic information for all participants. Data were tested for normality and homogeneity of variance (Shapiro–Wilk test (SW), α = 0.05). IGE = Idiopathic Generalised Epilepsy; SD = Standard Deviation

a)								
Patient	Age	Sex	Onset	Duration	Category	FH	PS	Seizures
1	34	F	2	32	REF	N	N	GTCS
2	23	F	14	9	REF	N	N	AS, MS
3	19	M	16	3	REF	Y	Y	GTCS
4	19	F	8	11	REF	Y	N	AS, GTCS
5	25	M	19	6	nonREF	N	N	MS
6	60	F	13	47	REF	Y	N	AS, GTCS
7	24	M	15	9	REF	Y	N	AS, MS, GTCS
8	21	F	15	6	REF	N	N	AS, MS, GTCS
9	32	F	23	9	REF	Y	N	MS, GTCS
10	38	M	18	20	REF	Y	N	GTCS
11	67	M	29	38	REF	N	N	AS, GTCS
12	46	F	7	39	REF	N	N	AS
13	20	M	8	12	REF	N	N	GTCS
14	24	F	13	11	REF	Y	N	MS, GTCS
15	35	M	6	29	REF	N	N	GTCS
16	18	M	14	4	REF	N	N	AS, GTCS
17	39	M	17	22	REF	Y	Y	GTCS
18	24	F	16	8	nonREF	N	N	AS, GTCS
19	21	M	16	5	REF	N	N	AS, MS, GTCS
20	36	F	17	19	REF	N	N	GTCS
21	31	F	15	16	REF	N	N	GTCS
22	31	F	16	15	REF	N	N	AS, MS, GTCS
23	23	M	16	7	nonREF	N	N	AS, GTCS
24	19	F	13	6	nonREF	Y	N	GTCS
25	58	F	15	43	REF	N	N	GTCS
26	18	F	15	3	nonREF	N	Y	AS, MS
27	22	M	2	20	nonREF	N	Y	AS, MS
28	24	M	13	11	REF	N	N	MS
29	56	F	3	53	nonREF	N	Y	AS
30	57	F	7	50	REF	N	N	AS, GTCS
31	33	M	7	26	nonREF	N	N	AS

b)				
	Patients with IGE	Controls	Normality	Stats
N	31	39		-
Sex (female/male)	17/14	24/15	SW = 0.626, P < 0.001	X^2^ = 0.319, P = 0.572
Mean age in years (SD), Range	32 (14.62), 18–67	32 (8.65), 21–60	SW = 0.898, P < 0.001	U = 502.00, n_1_ = 39, n_2_ = 31, P = 0.225
Mean age of Onset (SD)	14 (8.54)	-	-	-
Mean Duration in years (SD)	17.88 (15.41)	-	-	-
Controlled?	23 Refractory, 8 non-refractory	-	-	-

### MRI acquisition

Participants were scanned at the Department of Neuroradiology at the Walton Centre NHS Foundation Trust, Liverpool, UK on a 3 T GE Discovery MR 750 MRI scanner with a 32-channel head coil. For this study, two sequences were acquired in the same session: (1) T1-weighted fast spin-gradient (FSPGR) images with Phased Array Uniformity Enhancement (PURE) signal inhomogeneity correction (140 slices, TR = 8.2 ms, TI = 450 ms, TE = 3.22 ms, flip angle = 12, voxel size = 1 × 1 × 1 mm); (2) T1-FLAIR coronal images (52 slices, TR = N/A, TI = 920 ms, TE = 9.94 ms, flip angle = 111, voxel size = 0.4 × 0.4 × 3 mm).

### MRI processing

**Fig. 1:**
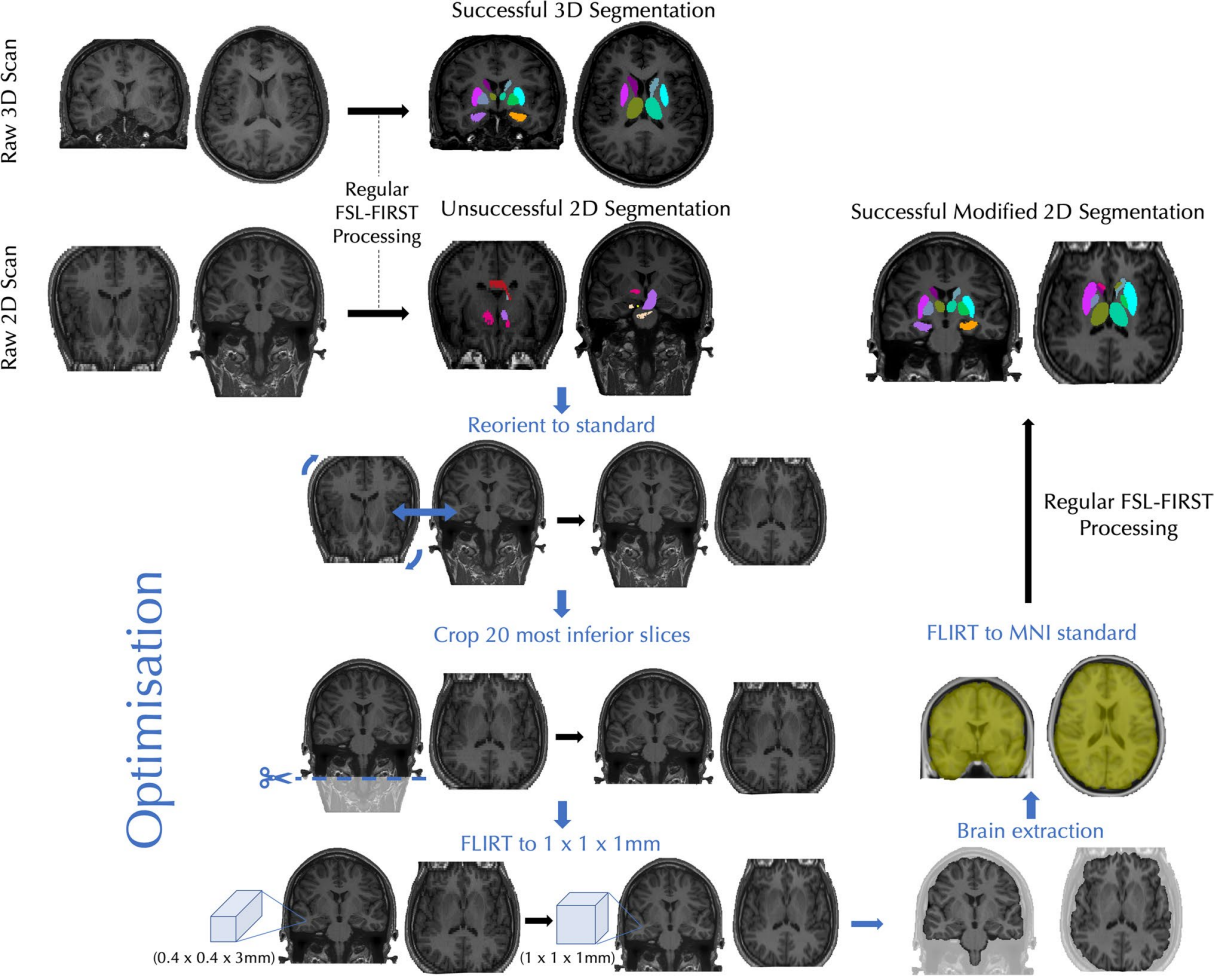
Pre-processing and segmentation of 3D and 2D MR images from an exemplar patient. Note the unsuccessful segmentation of the 2D image with regular processing but the success of segmentation following processing with the modified pipeline involving reorientation to standard, cropping of inferior slices, interpolation to an isotropic resolution of 1 × 1 × 1 mm, extraction of the brain and interpolation to the MNI standard brain

The FSL-integrated registration and segmentation toolbox (FIRST) software version 5.0.10 (http://fsl.fmrib.ox.ac.uk/fsl/fslwiki/first) was used for the automated segmentation and volume estimation of subcortical structures from 3 and 2D images. For 3D image analysis, the standard processing pipeline was followed as performed in previous studies of epilepsy [10–14]. Following the guidance of Amann et al., 2015 [7], it was necessary to modify the processing pipeline for analysis of the 2D images; a summary of the modified processing steps is provided in Fig. 1. Because of limited head coverage with some 2D sequences, acquisition may be rotated to maximise brain coverage. The first modification therefore included image rotation to a standard orientation. 3D images were not rotated. Image rotation led to partial inclusion of the brainstem and scalp. In order to best align rotated 2D images with the MNI standard template for image registration, the MNI standard brain had the twenty most inferior and superior slices removed. To aid the registration to the standard template, it was necessary to interpolate the 2D images to a 1 mm × 1 mm × 1 mm resolution. This was performed using FMRIB’s Linear Image Registration Tool (FLIRT) with a sinc function [33–35]. As part of the FSL package, the brain extraction tool (BET) [36] was used for the removal of non-brain voxels to aid registration to the standard template using normalized mutual information, which is more robust and computationally demanding compared to the default FLIRT registration options.

After registration, the left and right thalamus, caudate, putamen, pallidum, hippocampus and nucleus accumbens were segmented and the volume of each region of interest (ROI) was obtained. Although FIRST can also be used to segment the amygdala and brainstem, dimensions of some 2D images did not sample enough amygdala or brainstem tissue. This resulted in a failure to segment these regions when included, therefore the decision was made to not include these regions in the analysis of the present study. The images were visually inspected for quality control at various stages to ensure that the processing steps were being carried out successfully.

We additionally decreased the slice thickness of 2D images to determine whether measures of consistency between 2 and 3D volume estimations were impacted by an increasing slice thickness. In a randomly selected 50% of the cohort, images were averaged to generate a slice thickness of 6 mm using the FLIRT tool. These images were segmented using the above pipeline.

### Statistical analysis

Continuous data within the cohort were tested for normality using Shapiro–Wilk tests with an alpha-level of 0.05. Similarly to Amann et al. [7], 2D and 3D datasets were acquired in different orientations. Following the previous work, subcortical segmentations obtained from the 2D data were co-registered with 3D images prior to assessment of consistency between 2 and 3D segmentations of each ROI; this consistency was analysed using Dice coefficients (DCs) [37]. DCs represent percentage overlap values ranging from 0 (no overlap) to 1 (complete overlap). Cicchetti [38] and Zijdenbos et al. [39] have previously described the results from DC analysis that indicate poor agreement (< 0.2), fair agreement (0.2–0.4), moderate agreement (0.4–0.6), good agreement (0.6–0.8) and excellent agreement (0.8–1.00). Paired t-tests with Bonferroni correction were used to assess differences in volume between 2 and 3D image subcortical segmentations. In order to determine differences in subcortical volume between patients and controls, we used a multivariate ANOVA controlling for age, sex, and intracranial volume (ICV). ICV was calculated from 2 and 3D image segmentations using CAT (Computational Anatomy Toolbox) 12 (http://www.neuro.uni-jena.de/cat/) as has previously been done [40, 41]. Finally, we also investigated differences between patients that attained seizure freedom and those whose seizures persisted despite medical intervention, using multivariate ANOVA controlling for age, sex and ICV.

## Results

**Fig. 2:**
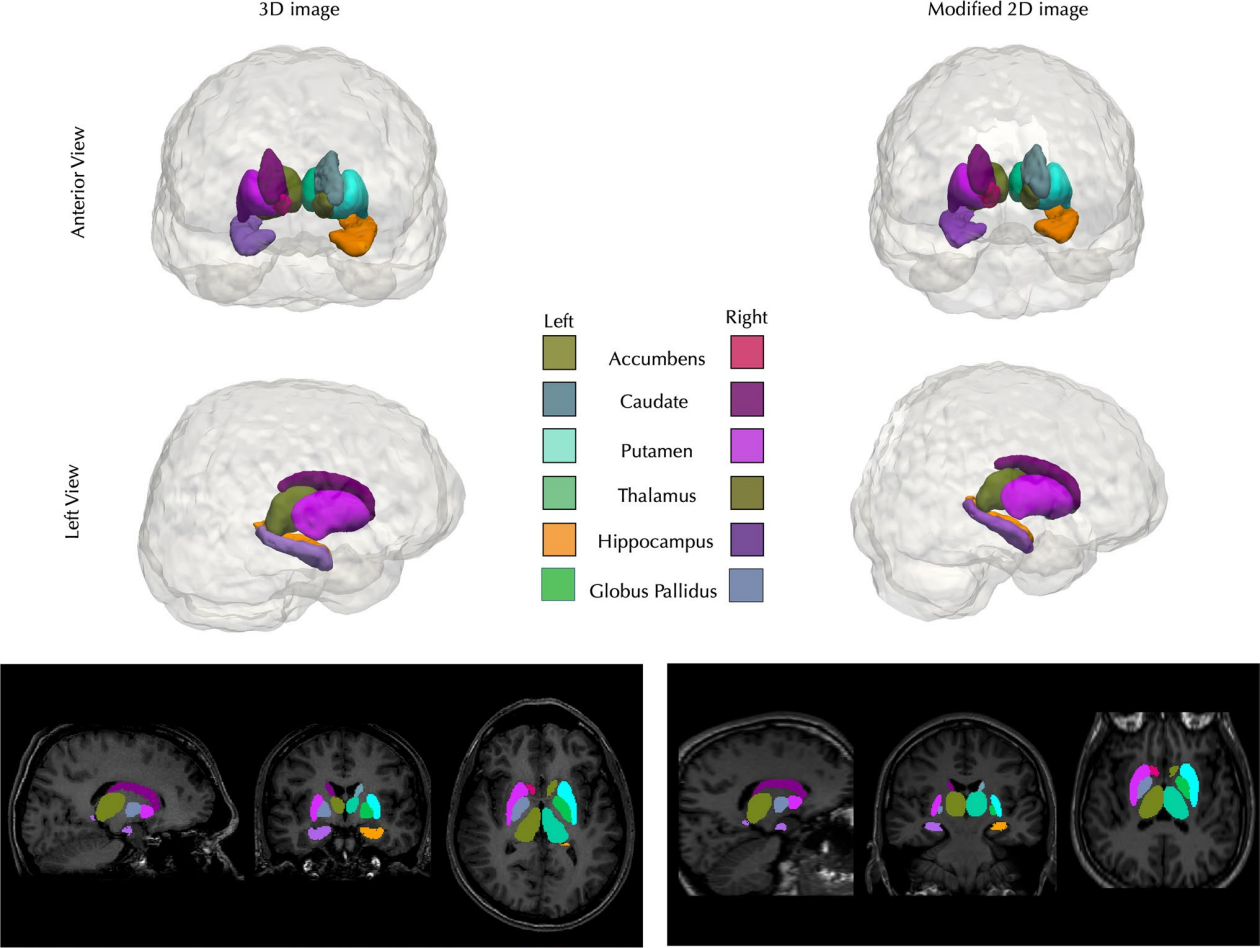
An exemplar 3D visualisation of the successful segmentations of the 3D (**a**) and 2D (**b**) images from the same patient. Note the Globus Pallidus is not visible on 3D renderings

**Fig. 3:**
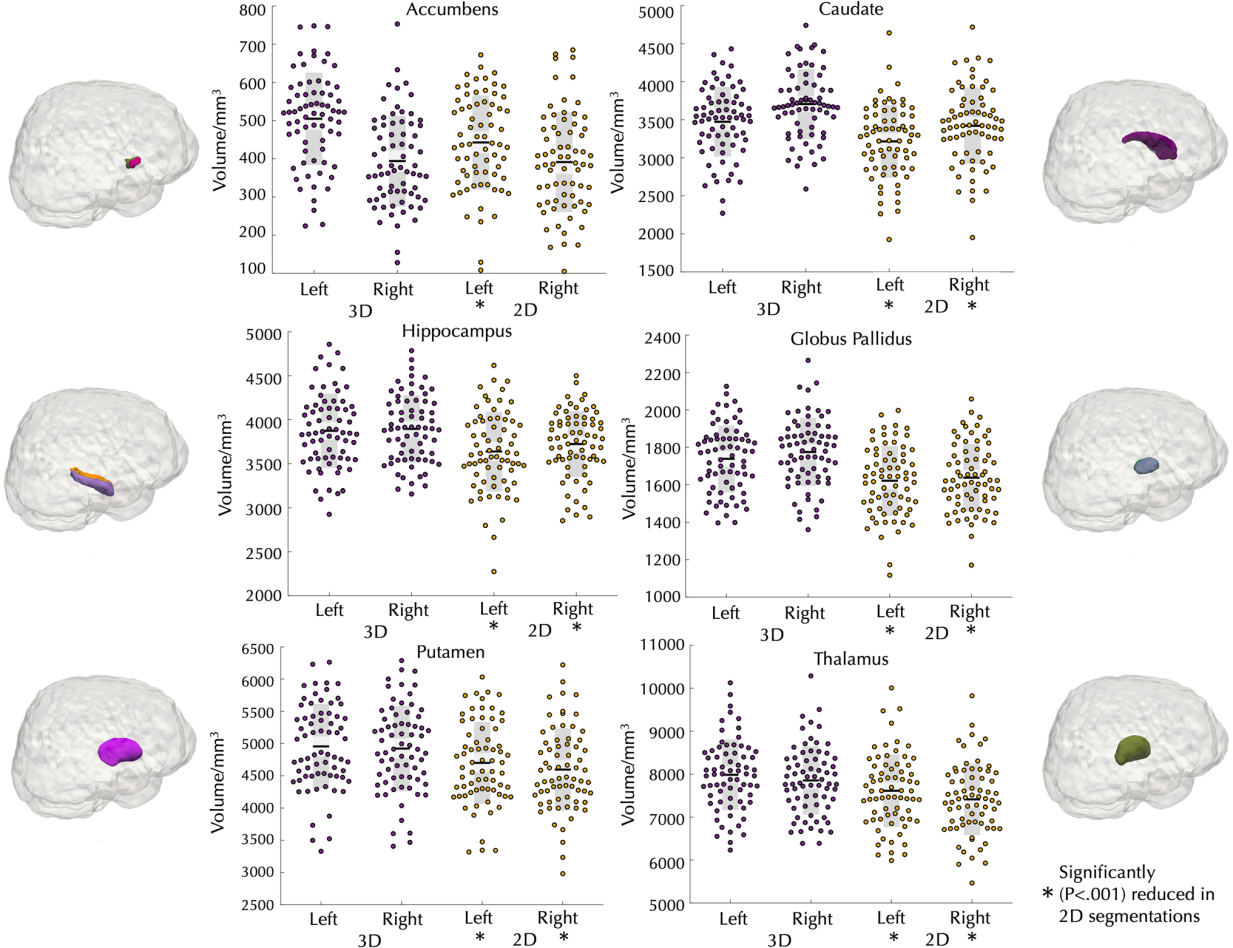
Differences between 3 and 2D images in volume estimation for each subcortical ROI. With the exception of the right accumbens, all ROIs had significantly reduced volume from 2D images relative to 3D images. (* = *P* < 0.001)

Normality tests revealed that most data were normally distributed (P > 0.05) with the exception of age and sex (P < 0.001). Successful segmentations were obtained for all patients from 3 and 2D images following processing with the modified pipeline. Exemplar comparative reconstructions of successful segmentations are shown in Fig. 2. No images showed segmentation inaccuracies when assessed visually. DCs demonstrated excellent agreement between all ROIs, with the right hippocampus having the lowest agreement (average DC = 0.86) and the left putamen having the highest agreement (average DC = 0.96) (See Table 2). Paired t-tests show that volumes derived from 3D images were slightly but consistently and significantly (P < 0.001) larger for each ROI, with the exception of the right accumbens, relative to the same ROIs obtained from 2D images (Table 3; Fig. 3).

**Table 2:** Average Dice coefficients for the comparison between 3 and 2D MRI images for each ROI

ROI	Whole average Dice coefficient	Average patient Dice coefficient	Average control Dice coefficient
Left accumbens	0.88	0.87	0.89
Right accumbens	0.90	0.88	0.92
Left caudate	0.91	0.91	0.91
Right caudate	0.91	0.91	0.92
Left hippocampus	0.88	0.88	0.89
Right hippocampus	0.86	0.86	0.87
Left globus pallidus	0.90	0.89	0.90
Right globus pallidus	0.89	0.89	0.90
Left putamen	0.96	0.95	0.96
Right putamen	0.95	0.95	0.95
Left thalamus	0.94	0.94	0.95
Right thalamus	0.94	0.94	0.95

**Table 3:** Descriptive and inferential statistics between subcortical volumes obtained from 3 and 2D images. SD = Standard deviation (Bonferroni corrected P-value = 0.004)

ROI	3D Mean (mm^3^)	2D Mean (mm^3^)	Mean difference	SD	t	Sig (2-tailed)
Left accumbens	504.79	442.71	62.07	113.43	4.58	< 0.001
Right accumbens	393.99	391.33	2.68	99.93	0.22	= 0.83
Left caudate	3473.37	3215.29	258.09	358.72	6.02	< 0.001
Right caudate	3706.59	3420.94	285.64	325.29	7.35	< 0.001
Left hippocampus	3873.57	3638.1	235.47	222.57	8.85	< 0.001
Right hippocampus	3895.11	3722.87	172.24	265.05	5.44	< 0.001
Left pallidum	1738.37	1621.13	117.24	120.92	8.11	< 0.001
Right pallidum	1774.5	1637.36	137.14	124.92	9.19	< 0.001
Left putamen	4955.54	4700.26	255.29	218.16	9.79	< 0.001
Right putamen	4921.91	4596.09	325.83	214.28	12.72	< 0.001
Left thalamus	7985.99	7613.83	372.16	239.65	12.99	< 0.001
Right thalamus	7854.33	7413.46	440.87	196.83	18.74	< 0.001

The DCs calculated for the volumes generated from images with an increased slice thickness showed a marked decrease in agreement with the 3D volumes. The lowest agreement was for the right hippocampus (average DC = 0.55) and the highest was the left putamen (average DC = 0.83) (See Table 4).

**Table 4:** Comparison of average Dice coefficients calculated for each ROI for both the 2D images and the 2D images with an increased slice thickness when compared to the ROIs of the 3D images

ROI	Original 0.4 mm × 0.4 mm × 3 mm average Dice coefficient	0.4 mm × 0.4 mm × 6 mm Average Dice coefficient (50%)
Left accumbens	0.88	0.67
Right accumbens	0.90	0.66
Left caudate	0.91	0.76
Right caudate	0.91	0.76
Left hippocampus	0.88	0.70
Right hippocampus	0.86	0.56
Left globus pallidus	0.90	0.73
Right globus pallidus	0.89	0.75
Left putamen	0.96	0.83
Right putamen	0.95	0.82
Left thalamus	0.94	0.82
Right thalamus	0.94	0.83

**Fig. 4:**
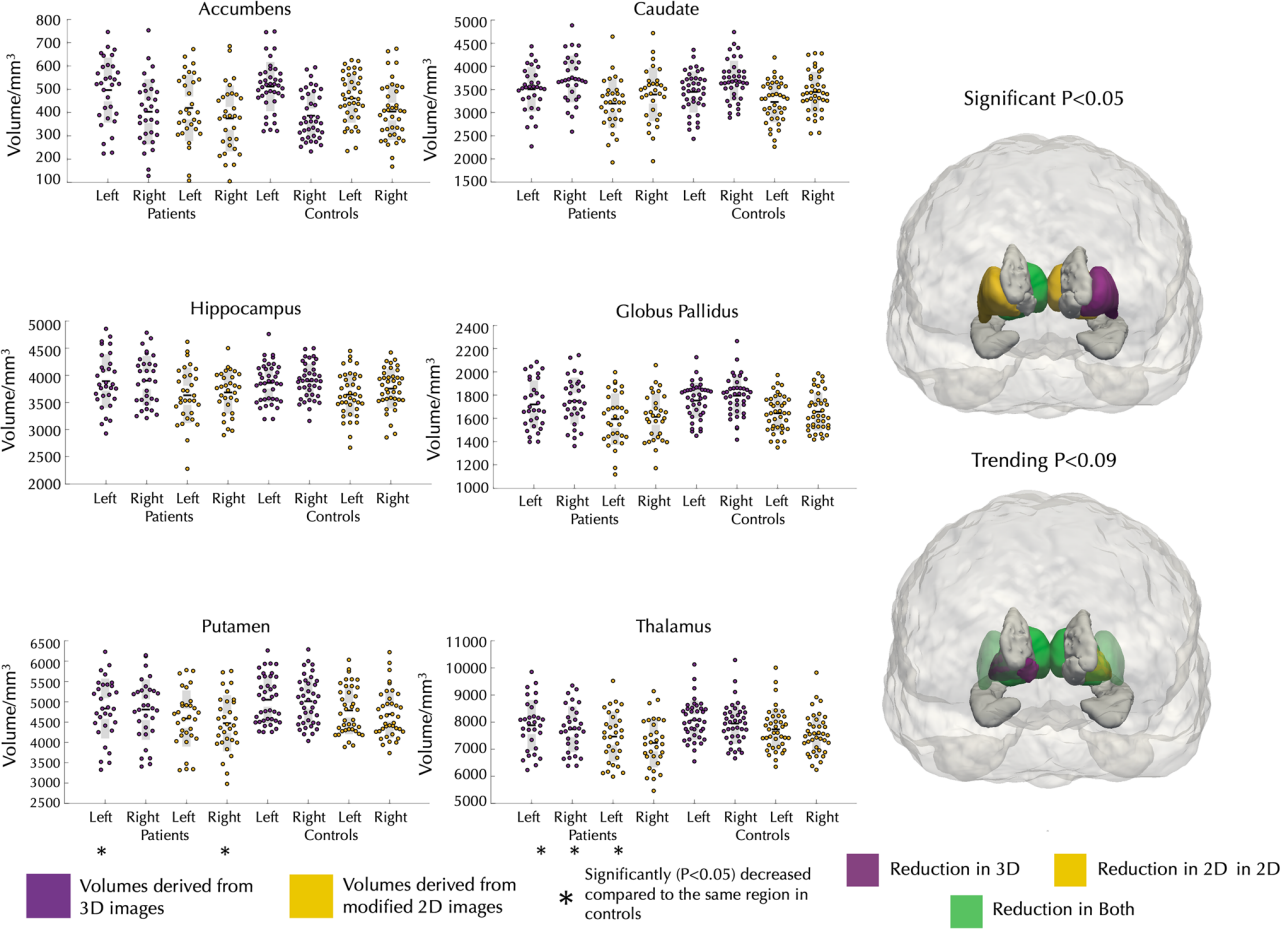
Volume differences between patients with IGE and healthy controls derived from 3 and 2D images. (* = *P* < 0.05)

Comparison of subcortical volumes between controls and patients with IGE using multivariate-ANOVA yielded similar results for analysis from 3 and 2D images (Fig. 4, Table 5). Analysis of the 3D volumes revealed that there were significantly lower volumes of the left putamen (P < 0.05) and right thalamus (P = 0.04) in patients compared to controls. Furthermore, there were strong trends for lower volume of left thalamus (P = 0.09), right putamen (P = 0.06) and right pallidum (P = 0.08) in patients relative to controls. Analysis of 2D volumes revealed significantly lower volumes of the right putamen (P = 0.04) and the left (P = 0.02) and right (P = 0.01) thalamus patients compared to controls, and strong trends for lower volume of the left putamen (P = 0.05) and left pallidum (P = 0.07) in patients compared to controls. There were no trends for volume differences of the left or right hippocampus, caudate or accumbens in patients relative to controls using 3D or 2D images.

**Table 5:** Descriptive and inferential statistics for comparison of subcortical volumes between controls and patients with IGE. *significant at p < 0.05, ^†^trend level, SD = Standard Deviation

		Patient volumes		Control volumes			
Structure	Approach	Mean	SD	Mean	SD	F	Sig
Left accumbens	3D	496.45	139.14	511.41	105.42	0.53	0.47
	2D	419.58	141.99	461.10	104.37	2.74	0.10
Right accumbens	3D	403.39	139.97	386.51	100.62	0.28	0.60
	2D	374.90	141.49	404.38	121.99	1.85	0.18
Left caudate	3D	3504.19	479.74	3448.87	444.18	0.23	0.64
	2D	3196.19	531.23	3230.46	438.01	0.20	0.66
Right caudate	3D	3727.55	502.46	3689.92	429.21	0.08	0.78
	2D	3392.58	575.71	3443.49	438.69	0.36	0.55
Left hippocampus	3D	3891.35	496.51	3859.44	358.26	0.08	0.78
	2D	3630.55	510.40	3644.10	406.68	0.11	0.75
Right hippocampus	3D	3900.61	454.88	3890.74	322.78	0.00	0.99
	2D	3683.68	387.82	3754.03	365.60	1.00	0.32
Left pallidum	3D	1719.81	206.57	1753.13	153.15	2.23	0.14
	2D	1593.19	220.29	1643.33	154.39	3.46	0.07^**†**^
Right pallidum	3D	1748.84	201.10	1794.90	165.37	3.16	0.08^**†**^
	2D	1613.03	198.47	1656.69	163.33	2.85	0.10
Left putamen	3D	4834.39	735.52	5051.85	578.76	4.09	0.05*
	2D	4588.90	690.08	4788.77	584.50	3.87	0.05^**†**^
Right putamen	3D	4808.23	742.27	5012.28	581.43	3.79	0.06^**†**^
	2D	4471.74	690.74	4694.92	582.24	4.64	0.04*
Left thalamus	3D	7881.71	930.50	8068.87	726.68	3.06	0.09^**†**^
	2D	7466.94	921.96	7730.59	771.69	5.68	0.02*
Right thalamus	3D	7736.52	839.58	7947.97	750.73	4.55	0.04*
	2D	7232.29	901.88	7557.46	744.38	8.35	0.01*

Comparison of volume differences between patients with refractory IGE, patients with non-refractory IGE and healthy controls revealed that there were significantly lower volumes in the right thalamus (P = 0.02) in refractory patients compared to controls when using 2D volumes. Strong trends for lower volumes in patients with refractory IGE compared to controls were found in the left (P = 0.06) and right (P = 0.09) putamen in analysis of 3D volumes and in the right accumbens (P = 0.06), left pallidum (P = 0.08), left (P = 0.08) and right (P = 0.07) putamen and left thalamus (0.06) in analysis of 2D volumes. The results of this analysis are summarised in Table 6.

**Table 6:** Descriptive and inferential statistics for comparison of subcortical volumes between patients who were medically refractory, those who attained seizure freedom and healthy controls. *significant at p < 0.05. ^†^trend level. SD = Standard Deviation

		Refractory patient volumes		Non-refractory patient volumes		Control volumes			
Structure	Approach	Mean	SD	Mean	SD	Mean	SD	F	Sig
Left accumbens	3D	480.57	127.19	542.13	170.04	511.41	105.42	0.39	0.68
	2D	404.65	139.55	462.50	149.51	461.10	104.37	1.50	0.23
Right accumbens	3D	394.61	137.59	428.63	153.25	386.51	100.62	0.14	0.87
	2D	381.78	144.26	355.13	140.66	404.38	121.99	3.03	0.06^**†**^
Left caudate	3D	3473.00	483.17	3593.88	490.19	3448.87	444.18	0.11	0.89
	2D	3117.96	481.65	3421.13	634.37	3230.46	438.01	0.49	0.62
Right caudate	3D	3717.17	448.66	3757.38	669.32	3689.92	429.21	0.27	0.76
	2D	3292.04	515.53	3681.63	675.67	3443.49	438.69	1.06	0.35
Left hippocampus	3D	3844.17	488.83	4027.00	526.48	3859.44	358.26	0.12	0.89
	2D	3567.83	496.62	3810.88	539.68	3644.10	406.68	0.28	0.76
Right hippocampus	3D	3887.26	471.29	3939.00	431.79	3890.74	322.78	0.15	0.86
	2D	3608.74	357.19	3899.13	414.85	3754.03	365.60	1.62	0.21
Left pallidum	3D	1703.87	196.40	1765.63	241.67	1753.13	153.15	1.15	0.32
	2D	1556.00	223.53	1700.13	182.57	1643.33	154.39	2.65	0.08^**†**^
Right pallidum	3D	1737.35	201.67	1781.88	209.35	1794.90	165.37	1.68	0.20
	2D	1596.09	174.02	1661.75	264.56	1656.69	163.33	1.41	0.25
Left putamen	3D	4714.09	680.33	5180.25	824.38	5051.85	578.76	2.89	0.06^**†**^
	2D	4479.30	621.12	4904.00	821.85	4788.77	584.50	2.59	0.08^**†**^
Right putamen	3D	4694.35	640.93	5135.63	951.36	5012.28	581.43	2.51	0.09^**†**^
	2D	4371.17	571.75	4760.88	942.67	4694.92	582.24	2.83	0.07^**†**^
Left thalamus	3D	7808.35	856.56	8092.63	1155.97	8068.87	726.68	1.53	0.23
	2D	7406.87	857.25	7639.63	1134.61	7730.59	771.69	2.97	0.06^**†**^
Right thalamus	3D	7676.43	785.40	7909.25	1017.99	7947.97	750.73	2.35	0.10
	2D	7165.96	827.87	7423.00	1129.55	7557.46	744.38	4.17	0.02*

## Discussion

The present study had two main objectives. Firstly, we sought to determine the consistency between volume estimation of subcortical structures obtained through a modified FSL-FIRST pipeline from 3 and 2D images in the same participants. We report that the average DC for all structures was above 0.85, representing an excellent level of agreement between 3 and 2D segmentations. Despite this consistency, volumes obtained from 2D images were lower than those from 3D images. DCs were reduced when 2D images were manipulated to double the slice thickness. Secondly, we sought to compare subcortical volume measures between patients with IGE and healthy controls using both 3D and 2D approaches. We report that both approaches demonstrate preferential lower volumes of the thalamus and putamen bilaterally, with some evidence of lower volume of the pallidum, in patients with IGE compared to controls. No effects were observed in the hippocampus, caudate or accumbens.

The results of the present study are in line with previous investigations that have modified the FSL-FIRST pipeline for successful segmentation of 2D images in patients with MS [7, 8]. In these studies, a certain proportion of the 2D images used failed to segment despite modification of the image pre-processing pipeline. In Amann et al. [7], consistency between 3 and 2D images was variable depending on the ROI examined, with some DCs falling below an acceptable agreement threshold. The present study has had an increased level of success in that both 3D and 2D images were successfully segmented for all participants with no processing failures or visible segmentation errors and that DCs demonstrated excellent agreement for all segmented regions. Other work [8] was unable to find consistent correlates between clinical measures of MS severity and volume estimates across 3D and 2D volumes using FSL-FIRST. They attribute this inconsistency to low intra-class correlation coefficients between 3 and 2D volumes derived from FIRST in their cohort — whereas we have found selective volume loss in the thalamus and putamen in both 3D and 2D volume analysis. The image contrasts used in this study are less similar to each other than the 2D fast spin echo and 3D MPRAGE contrasts used by Amann et al., [7]. This could account for the limited level of disagreement between 3 and 2D volume estimates and could be further reduced by using a more matched 2D sequence. However, calculated Dice coefficients still demonstrate excellent agreement between image types suggesting that inherent signal intensities and contrast differences between 3 and 2D volumes had limited impact on volume estimate, further supporting the pragmatic application of this subcortical volume estimation method to 2D MRI data.

The consistency between volume estimations observed in the present study could potentially be due to the higher in-plane resolution (0.4 mm × 0.4 mm) of the MR images in this study compared to previous studies [7, 8]. An important additional aspect is image slice thickness, as we have demonstrated, which may impact on segmentations from excessively thick slices obtained in non-specialist centres. We have shown that volumes derived from 2D images with increased slice thickness is associated with a marked reduction in agreement with volumes derived from 3D images. A conclusive evaluation of the influence of in-plane resolution on volume estimates would require a systemic approach that includes the acquisition of 2D and 3D images at various slice thicknesses and voxel dimensions. There is also the possibility to manipulate images, generate synthetic lower resolution data and assess subcortical segmentation performance, similar to what has recently been reported using Freesurfer [42].

Despite the consistency and apparent reliability of 2D volume estimations, caution should be urged when attempting to merge volumes obtained from 2 and 3D images in the same analysis given the consistently larger volumes obtained from 3D images. To confirm this, the 2D and 3D data for half of the participants were swapped and the analysis was performed again, which altered the findings (this data is included as supplementary material). The use of 2D MRI images opens up avenues for the inclusion of subcortical morphometric data in multi-institution research and clinical trials that may generate large datasets from routinely collected 2D MRI data. This offers the potential for important supplementary work in large scale epidemiological and clinical studies of treatment outcome in early epilepsy that have limited involvement of neuroimaging and subcortical morphometry by not including quantitative MRI information [5, 6]. In particular, combining subcortical volumes obtained from routinely acquired 2D MRIs with clinical outcome data from epilepsy trials such as SANAD (Standard and New Antiepileptic Drugs) [43] and MESS (Multicentre trial for early Epilepsy and Single Seizures) [44] may help identify prognostic biomarkers.

Patients with IGE showed evidence of lower volumes of the thalamus and putamen bilaterally compared to controls, and trends for smaller volume of the pallidum. These changes were observed using volumes obtained from both 3D and 2D images and fit with the well-known pattern of subcortical atrophy in patients with IGE. A lower volume in a particular brain region can be indicative of neuronal loss which could lead to altered function in that region. Thalamic atrophy has been previously reported in patients with IGE [2, 15–25, 45]. Involvement of the thalamus in IGE has long been implicated by electrophysiological studies of animal models reporting that abnormal oscillations in thalamocortical networks may play a crucial role in seizure onset [31, 46–48]. The thalamus, putamen and pallidum are involved either directly or indirectly in the thalamocortical networks implicated in the generation of generalised spike-wave discharges seen in IGE pathology [19]. Thalamic metabolites have been investigated using magnetic resonance spectroscopy (MRS) in patients with IGE and studies have reported reduced N-acetyl aspartate [49–51] and increased glutamate and glutamine [51] in patients relative to controls. These metabolite changes have been linked to thalamic atrophy [51]. Volume atrophy of the putamen in patients with IGE has also been reported in previous work [17–19, 26, 27]. It is believed that the putamen and other basal ganglia nuclei may have a role in the inhibition of epileptic seizures and abnormalities in putamen function affect the inhibitory power of this system [52]. Lower volume of the pallidum has also been previously reported in patients with IGE [18, 19]. The pallidum provides GABAergic input to the subthalamic nucleus and it has been reported that inhibition of GABA in the globus pallidus leads to the suppression of seizures [53]. Additionally, dopaminergic depletion in the pallidum can lead to the aggravation of absence seizures [53]. Volume loss in the pallidum seen in IGE could lead to altered GABAergic and dopaminergic functioning. It should be noted that despite thalamic and putamen, volume loss has been reported in numerous single site studies, the larger scale multi-site ENIGMA epilepsy studies report inconsistent findings. Only right thalamic atrophy was observed in IGE in the first ENIGMA Epilepsy volumetric study [44]. However, a more recent study from the ENIGMA Epilepsy consortium reported atrophy of the thalamus and putamen bilaterally and that this atrophy co-localised with highly interconnected subcortical hub regions [54].

Analysis of volume differences between patients with IGE who were medically refractory compared to those who were non-refractory and healthy controls, further reveals lower volumes in the right thalamus of refractory patients when using 2D volumes. Additionally, trends for lower volumes in refractory patients were found in regions similar to the pattern of atrophy established in the literature. Further investigation into these differences with this method could provide an approach to clinically distinguish between patients who will attain seizure freedom and those will remain refractory.

There are some limitations to this study. All 2D image data were obtained using the same protocol on the same MRI scanner. Therefore, caution should be taken when combining multiple acquisition protocols in the same analysis as we have not examined the impact of this on volume estimation. Differences in acquisition protocol parameters and scanner models across different institutions may be harmonised using methods such as ComBat [55], which has been used in current multi-centre epilepsy neuroimaging research [56, 57]. Furthermore, some subcortical findings were trending towards significance. It is possible that with a larger cohort size that the trend towards lower volumes seen in patients would reach statistical significance. This investigation is also limited as FIRST is only able to segment the subcortical region as opposed to Freesurfer (https://surfer.nmr.mgh.harvard.edu/) which can perform automatic whole-brain segmentation. Agreement between 2 and 3D MR images has been found to be respectable when segmentation is performed using Freesurfer [8]. This previous study indicated that, while segmentation of the subcortical structures is possible, cortical thickness estimates from 2D images may be unreliable and as such recommends a focus on subcortical investigations in modified 2D MR data. Additionally, Freesurfer is time consuming and often requires manual correction of segmentation. Nevertheless, the primary goal of the present study was to examine the consistency between 2 and 3D acquisitions for the identification of subcortical changes in IGE. We have provided evidence indicating high levels of consistency.

## Conclusion

We have demonstrated that reliable volume estimation of subcortical structures can be obtained from automatic segmentation from 2D MRI data using a modified FSL-FIRST pipeline. Volume estimation is most reliable from images with higher in-plane resolution (e.g. ≤ 1 mm × 1 mm) and moderate slice thickness (e.g. ≤ 3 mm). We further demonstrate that both 2D and 3D image segmentations provide evidence of thalamic and putamen atrophy in patients with IGE. Given that the software is freely available, this approach may be useful for large, multi-centre clinical trials that only have access to 2D MRI data acquired in non-specialist hospitals in context of routine clinical evaluation of patients.

## Supplementary Information

Below is the link to the electronic supplementary material.

Supplementary file1 (DOCX 15 KB)

## Data Availability

The data that support the findings of this study are available on request from the corresponding author. The original data are not publicly available due to ethical restrictions.

## Code availability

The code that supports the findings of this study is available on request from the corresponding author.
