# Supplementary material for: Automated subcortical volume estimation from 2D MRI in epilepsy and implications for clinical trials
Source: Neuroradiology. 2021 Oct 18;64(5):935–47. doi: 10.1007/s00234-021-02811-x (PMC9005416; doi:10.1007/s00234-021-02811-x)
Supplement: Supplementary file 1 — Supplementary file1 (DOCX 15 KB) [file 234_2021_2811_MOESM1_ESM.docx]

**Supplementary Table: The change in significant results that results from swapping the 3D and 2D images for a random selection of half of the participants in the analysis of differences between patients and controls; demonstrating that caution should be used when mixing 2D and 3D MRI data in the same analysis.**

|  |  | Original Results | Random Split |
| --- | --- | --- | --- |
| Structure | Approach | Sig. | Sig. |
| Left Accumbens | 3D | 0.47 | 0.10 |
|  | 2D | 0.10 | 0.48 |
| Right Accumbens | 3D | 0.60 | 0.27 |
|  | 2D | 0.18 | 0.73 |
| Left Caudate | 3D | 0.64 | 0.97 |
|  | 2D | 0.66 | 0.97 |
| Right Caudate | 3D | 0.78 | 0.88 |
|  | 2D | 0.55 | 0.61 |
| Left Hippocampus | 3D | 0.78 | 0.67 |
|  | 2D | 0.75 | 0.74 |
| Right Hippocampus | 3D | 0.99 | 0.35 |
|  | 2D | 0.32 | 0.92 |
| Left Pallidum | 3D | 0.14 | 0.07 |
|  | 2D | 0.07 | 0.20 |
| Right Pallidum | 3D | 0.08 | 0.12 |
|  | 2D | 0.10 | 0.14 |
| Left Putamen | 3D | 0.05 | 0.02 |
|  | 2D | 0.05 | 0.14 |
| Right Putamen | 3D | 0.06 | 0.03 |
|  | 2D | 0.04 | 0.09 |
| Left Thalamus | 3D | 0.09 | 0.04 |
|  | 2D | 0.04 | 0.07 |
| Right Thalamus | 3D | 0.02 | 0.02 |
|  | 2D | 0.01 | 0.03 |
